# Differentiation of Early Sacroiliitis Using Machine-Learning- Supported Texture Analysis

**DOI:** 10.3390/diagnostics15020209

**Published:** 2025-01-17

**Authors:** Qingqing Zhu, Qi Wang, Xi Hu, Xin Dang, Xiaojing Yu, Liye Chen, Hongjie Hu

**Affiliations:** 1 Department of Radiology, Sir Run Run Shaw Hospital, Zhejiang University School of Medicine, Hangzhou 310016, China; 3416243@zju.edu.cn (Q.Z.); y223180029@zju.edu.cn (Q.W.); zjuhuxi@zju.edu.cn (X.H.); 3307009@zju.edu.cn (X.Y.); orange2139@163.com (L.C.); 2 Department of Rheumatology, Sir Run Run Shaw Hospital, Zhejiang University School of Medicine, Hangzhou 310016, China; 3415027@zju.edu.cn

**Keywords:** texture analysis, sacroiliitis, magnetic resonance imaging

## Abstract

**Objectives:** We wished to compare the diagnostic performance of texture analysis (TA) against that of a visual qualitative assessment in identifying early sacroiliitis (nr-axSpA). **Methods:** A total of 92 participants were retrospectively included at our university hospital institution, comprising 30 controls and 62 patients with axSpA, including 32 with nr-axSpA and 30 with r-axSpA, who underwent MR examination of the sacroiliac joints. MRI at 3T of the lumbar spine and the sacroiliac joint was performed using oblique T1-weighted (W), fluid-sensitive, fat-saturated (Fs) T2WI images. The modified New York criteria for AS were used. Patients were classified into the nr-axSpA group if their digital radiography (DR) and/or CT results within 7 days from the MR examination showed a DR and/or CT grade < 2 for the bilateral sacroiliac joints or a DR and/or CT grade < 3 for the unilateral sacroiliac joint. Patients were classified into the r-axSpA group if their DR and/or CT grade was 2 to 3 for the bilateral sacroiliac joints or their DR and/or CT grade was 3 for the unilateral sacroiliac joint. Patients were considered to have a confirmed diagnosis if their DR or CT grade was 4 for the sacroiliac joints and were thereby excluded. A control group of healthy individuals matched in terms of age and sex to the patients was included in this study. First, two readers independently qualitatively scored the oblique coronal T1WI and FsT2WI non-enhanced sacroiliac joint images. The diagnostic efficacies of the two readers were judged and compared using an assigned Likert score, conducting a Kappa consistency test of the diagnostic results between two readers. Texture analysis models (the T1WI-TA model and the FsT2WI-TA model) were constructed through feature extraction and feature screening. The qualitative and quantitative results were evaluated for their diagnostic performance and compared against a clinical reference standard. **Results:** The qualitative scores of the two readers could significantly distinguish between the healthy controls and the nr-axSpA group and the nr-axSpA and r-axSpA groups (both *p* < 0.05). Both TA models could significantly distinguish between the healthy controls and the nr-axSpA group and the nr-axSpA group and the r-axSpA group (both *p* < 0.05). There was no significant difference in the differential diagnoses of the two TA models between the healthy controls and the nr-axSpA group (AUC: 0.934 vs. 0.976; *p* = 0.1838) and between the nr-axSpA and r-axSpA groups (AUC: 0.917 vs. 0.848; *p* = 0.2592). In terms of distinguishing between the healthy control and nr-axSpA groups, both the TA models were superior to the qualitative scores of the two readers (all *p* < 0.05). In terms of distinguishing between the nr-axSpA and r-axSpA groups, the T1WI-TA model was superior to the qualitative scores of the two readers (*p* = 0.023 and *p* = 0.007), whereas there was no significant difference between the fsT2WI-TA model and the qualitative scores of the two readers (*p* = 0.134 and *p* = 0.065). **Conclusions:** Based on MR imaging, the T1WI-TA and fsT2WI-TA models were highly effective for the early diagnosis of sacroiliac joint arthritis. The T1WI-TA model significantly improved the early diagnostic efficacy for sacroiliac arthritis compared to that of the qualitative scores of the readers, while the efficacy of the fsT2WI-TA model was comparable to that of the readers.

## 1. Introducion

Axial spondyloarthritis (axSpA) is a chronic inflammatory disease affecting the spine and sacroiliac joints. Its diagnosis is often delayed for years due to the nonspecific nature of back pain, its primary symptom. AxSpA includes two subtypes: nonradiographic (nr-axSpA), which does not meet the modified New York criteria on X-rays, and radiographic (r-axSpA or ankylosing spondylitis), which fulfills these criteria, showing advanced structural changes in the sacroiliac joints.

Imaging for axial spondyloarthritis (axSpA) focuses on early sacroiliitis detection to enable timely treatment. The 2009 ASAS classification identified bone marrow edema (BME) as a sensitive MRI feature for the classification criteria, enhancing the diagnostic reliability for nr-axSpA by surpassing the traditional radiographic limitations and emphasizing the critical role of MRI in improving diagnostic precision [1]. However, due to the heterogeneity of axSpA, the imaging features of BME are not sufficient for its diagnosis, leading to the 2019 ASAS update, which emphasized the importance of incorporating structural lesions, such as erosions and fat signal changes, alongside BME to further enhance the diagnostic reliability for nonradiographic axial spondyloarthritis (nr-axSpA) [2]. In 2024, a collaborative study by ASAS (the Assessment of SpondyloArthritis International Society) and SPARTAN proposed a standardized MRI image acquisition protocol (IAP) for sacroiliitis [3]. This protocol highlights the critical importance of T1-weighted imaging (T1WI) sequences in detecting structural damage, ensuring consistent and comprehensive assessments of both active inflammation and structural changes to advance the diagnostic precision. Nevertheless, even for radiologists, identifying these imaging features at the nr-axSpA stage can be challenging. Therefore, a method that evaluates quantitative features may offer the best accuracy in predicting nr-axSpA.

Texture analysis (TA), a computer-aided imaging technique, has shown great potential in extracting quantitative features from medical images, capturing subtle structural changes that may be overlooked during conventional evaluations. TA has been widely applied in musculoskeletal imaging, including the differentiation of benign and malignant tumors [4], as well as the assessment of cartilage [5] and soft tissue structures [6]. However, its application in the diagnosis of early sacroiliitis remains underexplored.

This study aims to bridge the diagnostic gap for nr-axSpA by utilizing a texture analysis (TA) to quantitatively analyze MRI scans of the sacroiliac joint. Specifically, we focus on oblique coronal T1-weighted imaging (T1WI) and oblique fluid-sensitive, fat-saturated T2-weighted imaging (Fs-T2WI) to capture both structural changes and acute inflammation. By integrating TA with conventional MRI, we hypothesize that the TA can enhance the diagnostic accuracy, improve the early detection of nr-axSpA, and provide a robust alternative to traditional qualitative radiologist-based assessments. This study further explores the MRI-based texture features by comparing radiologist assessments with TA-enhanced models to validate the clinical utility of this approach.

## 2. Materials and Methods

### 2.1. Subjects

This study was approved by the Ethics Committee of Sir Run Run Shaw Hospital, Zhejiang University School of Medicine, and informed consent was waived due to the retrospective and anonymized nature of this study. In total, 471 patients who visited the Rheumatology Department from Jan. 2015 to Jan. 2018 were included. All patients received 3T sacroiliac joint and lumbar spine examinations and X-rays and/or computed tomography (CT) scans within one week of the MRI. All patients were diagnosed with axSpA after over 5 years of clinical and imaging follow-up.

**Figure 1:**
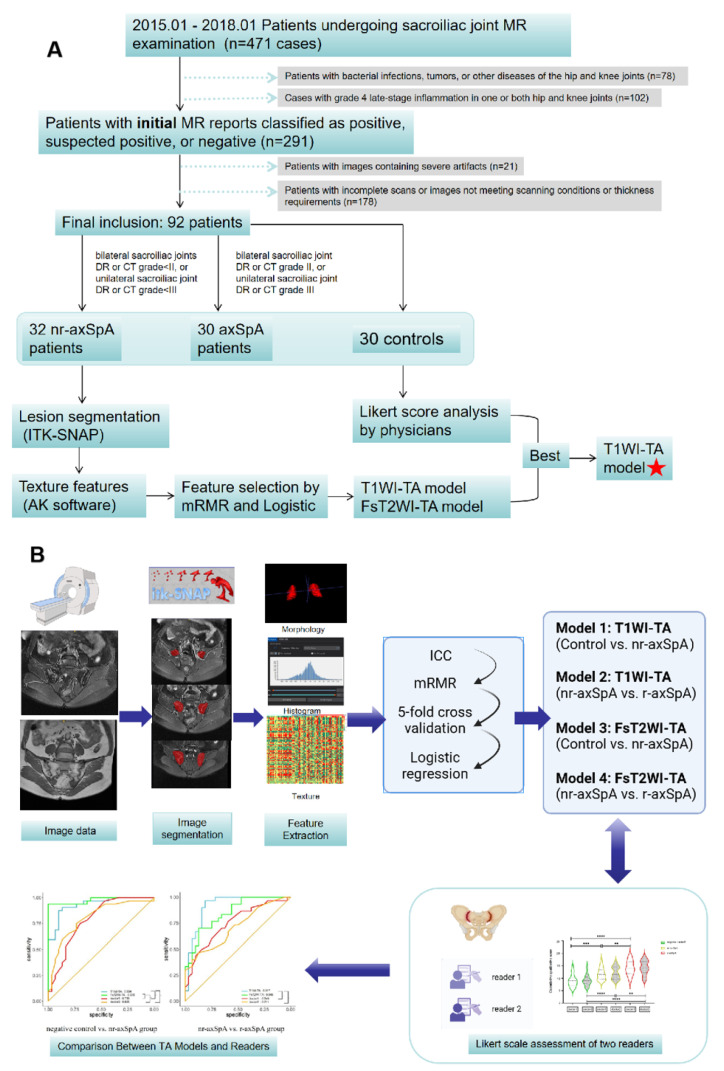
Patient selection and study design. (**A**) Flow diagram of the patient selection process; (**B**) workflow for TA feature extraction, model building, and comparison between readers and TA models. LR = logistic regression; ICC = intraclass correlation coefficient; TA = texture analysis; mRMR = minimum redundancy maximum relevance.

A total of 30 patients who visited the hospital for other reasons were included in the control group and diagnosed with normal sacroiliac joints during the clinical follow-up. The patients were screened to exclude the possibility of a transition to rheumatoid disorder in order to confirm the diagnosis. The ASAS exclusion criteria are as follows: a history of arthritis, enteritis, and dactylitis; a family history of SpA; positive HLA-B27; pathological ESR or CRP levels; psoriasis, inflammatory bowel disease, or uveitis; and a good response to treatment with nonsteroidal antirheumatics (NSARs). Therefore, rheumatologists ruled out any underlying rheumatism conditions to establish a healthy control cohort. The patient recruitment pathway is shown in Figure 1A.

### 2.2. Dataset Description

The dataset includes 92 subjects, comprising 62 cases of axSpA (32 nr-axSpA and 30 r-axSpA) and 30 cases in the control group. MRI scans from these subjects were used to create a dataset comprising Fs-T2WI and T1WI sequences. Each case included Fs-T2WI and T1WI sequences, with 19 to 20 slices per sequence, resulting in a total of 3628 images. The dataset was categorized into three groups—nr-axSpA, r-axSpA, and control—with 1180 images in the control group, 1280 in the nr-axSpA group, and 1,168 in the r-axSpA group. Details of the dataset are provided in Supplementary Material Data S1, Table S1 and Figure S1.

### 2.3. Data Collection

In this study, a GE 3.0 T MRI scanner (GE, MR Signa HDx 3.0T MRI Scanner, Chicago, IL, USA) with an 8-channel body phased-array (BPA) receive coil setup was used. During the scan, the subjects were in the supine position, and their feet were scanned first. All subjects underwent T1WI (oblique coronal), T1WI (axial), Fs-T2WI (oblique coronal), and Fs-T2WI (oblique axial) scans. Fast spin echo (fsE) was used for conventional sacroiliac joint sequencing. The scanning parameters in the oblique coronal T1WI process were as follows: repetition time (TR): 720 ms; echo time (TE): 7.3–22.0 ms; field-of-view (FOV): 456 mm × 456 mm; matrix: 320 × 224; number of slices: 21; slice thickness: 4 mm; slice spacing: 1.0 mm. The T1WI (axial) scanning parameters were as follows: TR: 500 ms; TE: 9.8–29.5 ms; FOV: 432 mm × 432 mm; matrix: 352 × 192; number of slices: 20; slice thickness: 5 mm; slice spacing: 1.0 mm. The coronal Fs-T2WI scanning parameters were as follows: TR: 3675 ms; TE: 85 ms; matrix: 320 × 224; number of slices: 19–20; slice thickness: 4 mm; slice spacing: 1.0 mm. The oblique axial Fs-T2WI scanning parameters were as follows: TR: 3920 ms; TE: 85 ms; matrix: 320 × 224; number of slices: 19–20; slice thickness: 5 mm; slice spacing: 1.0 mm. The MRI scanning parameters and the orientation of the oblique positioning lines for the sacroiliac joints are detailed in Supplementary Table S2 and Supplementary Figure S3.

### 2.4. Data Processing

#### 2.4.1. Analysis of Clinical Records

For the 30 subjects in the control group, there were 60 sacroiliac joints. The group included 17 males and 13 females (age: 18–44 years; average age: 31.00 ± 8.63 years). All subjects had no previous trauma, no infection, no history of surgery on the sacroiliac joints, no history of other chronic diseases with long-term drug treatment, no pain or morning stiffness of the bilateral sacroiliac joints, no inflammation of the peripheral joints or extra-articular organs, and no difficulties in their activities. There were 124 sacroiliac articular surfaces in the 62 patients in the axSpA group. Among them, 39 were males, and 23 were females (age: 18–44 years; average age: 31.18 ± 8.80 years). The diagnosis of axSpA was based on the 2009 ASAS classification criteria [1] (ruling out other sacroiliac joint diseases or treatment history). Based on the X-ray or CT scans (Supplementary Data S2: The radiological classification standards) within 7 days before or after MR, the nr-axSpA group included 32 cases, and the r-axSpA group included 30 cases. According to the clinical and follow-up diagnosis results, the subjects were divided into three groups: the negative control group (30 cases), the nr-axSpA group (32 cases) (bilateral sacroiliac joints of a DR or CT grade < 2 or unilateral sacroiliac joint of a DR or CT grade < 3), and the r-axSpA group (30 cases) (bilateral sacroiliac joint of DR or CT grade 2 or unilateral sacroiliac joint of DR or CT grade 3). Cases with unilateral or bilateral grade 4 sacroiliitis were not included in this study.

#### 2.4.2. Likert Scale Assessment of Two Readers

Based on the oblique coronal T1WI and Fs-T2WI MR imaging results, the MR findings of acute-phase BME showed hyperintensity in the Fs-T2WI sequence, and the corresponding area in T1WI was an edema zone with a blurred contour. Structural changes in the sacroiliac joint included fat metaplasia under the articular surface, hyperintensity in T1WI, and hypointensity in the corresponding area in fsT2WI; fat metaplasia in an erosion cavity (also known as “backfill”), subchondral bone worm-like destruction, and limited bone defects under the articular surface in T1WI and Fs-T2WI; and sclerosis and a hypointensity zone under the articular surface in both Fs-T2WI and T1WI. In terms of the formation of a local bone bridge/ankylosis formation, the Fs-T2WI and T1WI showed local connections between the sacrum and the ilium. The T1WI and Fs-T2WI DICOM images from 92 cases were used for the analysis. The typical features are highlighted in Supplementary Figure S4: Lesions of sacroiliitis.

The MR images of the sacroiliac joints were independently assessed by two readers (reader 1 has 7 years of experience in musculoskeletal disease diagnosis and is trained in sacroiliac joint imaging evaluation, and reader 2 has 6 years of experience in musculoskeletal disease diagnosis). The clinical diagnosis results were blinded to the readers. Based on the oblique coronal T1WI and Fs-T2WI sequences for each patient, the readers first classified the patients into the control group, the nr-axSpA group, or the r-axSpA group. Then, according to the six signs of sacroiliitis, including BME and structural changes, fat metaplasia, backfill, erosions, subchondral sclerosis, and bone bridges, the left and right sacroiliac joints were rated on a 4-point Likert scale (0: normal appearance; 1: mild alteration; 2: moderate alteration; and 3: severe alteration). The score for each sacroiliac joint was calculated by summing the score for each sign. Prior to scoring, the two readers were trained on images that were not related to this study. The X-ray and/or CT classification of 62 patients was performed according to the modified New York criteria.

#### 2.4.3. Manual Delineation of the Lesion Features and Extraction of the Texture Features

The ITK-SNAP software, Version 3.8, USA was used to manually delineate the two image sequences (oblique coronal T1WI and Fs-T2WI) of the sacroiliac joints of each patient. Bilateral axSpA lesions were located in the subchondral bone marrow area with or without destruction of the articular cartilage and the subchondral bone. Therefore, the lesion area was outlined as the statistically measured region of interest (ROI), and the joint space between the sacroiliac cartilage and the bone marrow area under the joint was outlined simultaneously. The para-articular line included the sacroiliac joint space. Supplementary Figure S2 illustrates the segmentation process for the sacroiliac joint lesions. For the control group, the anatomical-based ROI was manually outlined, including the sacroiliac joint space and 2–3 mm below the articular surface, and blood vessels and tendons were avoided. Any confounding anatomical factors, such as blood vessels, nerves, or muscles, were excluded from the outlined area. The delineation of negative cases was tested using many images that were not related to this study, and in those cases, the largest possible size that met all of the criteria was used. In the TA software (GE AK software, version 1.0.3, USA), the images were normalized based on the average value of the histogram and three standard deviations (SDs) to eliminate the technical differences between and within scans. The patient information was randomly assigned to two additional radiologists with an average of 4 years of experience in osteomuscle diagnostics for delineation of the ROIs, and the annotation process was based on the open-source software ITK-SNAP. The consistency evaluation of the feature extraction is detailed in Supplementary Data S3.

The artificial intelligence software AK was developed by GE Life Sciences for feature extraction and analysis, and it can be combined with ITK-SNAP to obtain three-dimensional images. The original T1WI and Fs-T2WI sequences and the ROI images were imported into the AK software in batches. The ROI feature extraction parameters were selected in the data selection window, and seven types of TA features were then selected in the AK software. In the gray-level co-occurrence texture matrix (GLCM), the gray-level size zone matrix (GLSZM), the gray-level run-length matrix (GLRLM), the neighborhood gray-tone difference matrix (NGTDM), and the gray-level dependence matrix (GLDM), some commonly used statistical methods were used to quantify the distribution of the voxel intensity in the MR images to obtain descriptive features; among these, 109 original features were chosen for subsequent screening.

#### 2.4.4. Texture Feature Screening

**Figure 2:**
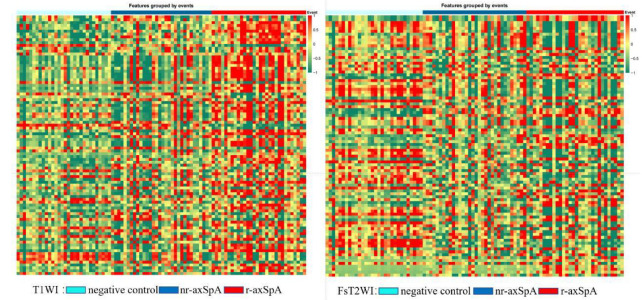
T1WI texture feature and T2WI texture feature heat maps.

Due to the large number of texture parameters, it was necessary to screen the feature parameters before the analysis to reduce the redundancy of the feature data and to thereby select the most distinguishable features between the control group and the nr-axSpA group and between the nr-axSpA group and the r-axSpA group, i.e., to determine the best model for nr-axSpA diagnosis. The R software (v4.3.1, R Foundation for Statistical Computing, Vienna, Austria) was used to reduce the extracted texture features. First, parameters with an intraclass correlation coefficient (ICC) of less than 0.8 were excluded. As a result, 96 features were retained in the T1WI sequences, and 90 features were retained in the T2WI sequences. The heat map (Figure 2) shows that the 96 T1WI features are different between the control group and the nr-axSpA group, and the difference was significant between the nr-axSpA group and the r-axSpA group; the 90 T2WI features were significantly different between the control group and the nr-axSpA group, yet the difference was not significant between the nr-axSpA group and the r-axSpA group. Next, five-fold cross-validation was repeated 10 times to obtain the average area under the curve (AUC). Specifically, the subjects were randomly divided into five groups, four of which were included in the training set and one of which was used as the validation set. The max relevance and min redundancy (mRMR) screening method was used to find the set of features in the original feature set with the maximum relevance and the minimum redundancy. All highly redundant features, i.e., features with a Pearson’s correlation coefficient of 0.75 or higher, were excluded. A total of five types of features were obtained for the two sequences in this study. The texture parameters obtained after mRMR dimension reduction were used as the texture feature modeling parameters, and logistic regression was used to predict the probability of an event. Moreover, the Akaike information criterion (AIC) backward method was used to prevent overfitting, and the median model in the 50 verification tests was selected as the final model.

#### 2.4.5. Establishment of the TA Models

Four texture feature models were established according to the source of the selected features. In Model 1, a T1WI sequence is used to identify negative and nr-axSpA cases; in Model 2, a T1WI sequence is used to identify nr-axSpA and r-axSpA cases; in Model 3, a T2WI sequence is used to identify negative and nr-axSpA cases; and in Model 4, a T2WI sequence is used to identify nr-axSpA and r-axSpA cases. The model performance was evaluated using the accuracy (Acc), sensitivity (Sen), specificity (Spe), positive predictive value (PPV), and negative predictive value (NPV), and the stability of the model was evaluated using the AUC.

#### 2.4.6. Statistical Analysis

The GraphPad Prism 9.4.1 software, which is based on equal-division estimation with qualitative cumulative scores, was used for the statistical analysis. The weighted kappa (κ) was used to determine the observer agreement for all qualitative variables. According to Landis and Koch [7], the agreement was classified as follows: poor agreement (κ < 0), slight agreement (0 < κ < 0.2), mild agreement (0.21 < κ < 0.4), moderate agreement (0.41 < κ < 0.6), considerable agreement (0.61 < κ < 0.8), and almost perfect agreement (0.81 < κ). The Paired Samples t-test was used to analyze the interobserver agreement between the two readers on the average scores for all of the qualitative parameters (BME, joint effusion, bone destruction, etc.) of the sacroiliac joints in the control group, the nr-axSpA group, and the r-axSpA group. In addition, the difference in the average scores of the qualitative parameters between the nr-axSpA group and the r-axSpA group was also analyzed by the senior reader.

An analysis of variance (ANOVA) was used to evaluate the differences in the cumulative scores of the qualitative parameters in the control group, the nr-axSpA group, and the r-axSpA group. The diagnostic indices between the control group and the nr-axSpA group and between the nr-axSpA group and the r-axSpA group were compared by the two readers. The AUC of the receiver operating characteristic (ROC) curve of the two readers was calculated to evaluate the diagnostic performance of the cumulative qualitative scores.

Lastly, an ROC analysis was carried out between the TA results and the results of the qualitative analysis. The statistical significance was set to *p* < 0.05. The workflow for the texture analysis, including the feature extraction, model building, and comparison between the radiologists and TA models, is shown in Figure 1B.

## 3. Results

### 3.1. Baseline Data

In total, 92 participants were enrolled in this study. A total of 62 cases were enrolled in the axSpA group, including 39 males and 23 females (age: 18–62 years; average age: 32.42 ± 10.94 years). Thirty cases were included in the negative control group (age: 18–55 years; median age: 30.5 years; average age: 31 ± 8.63 years).

There was no significant age difference between the groups (*p* = 0.59).

### 3.2. Qualitative Analysis

Reader 1 gave a qualitative score to each feature of the MRI images, and the scores and numbers of features for each group are shown in Table 1. There was no significant difference in the scores for the various signs between the negative control group and the nr-axSpA group. Significant differences in the Likert scores for BME (*p* < 0.01) and subchondral bone erosion (*p* < 0.05) between the nr-axSpA group and the r-axSpA group were identified by reader 1. Reader 2’s scoring table is provided in Supplementary Table S3.

The diagnostic agreement between reader 1 and reader 2 is shown in Table 1. The highest agreement was reached for BME (κ = 0.652), moderate agreement was reached for erosion and fat metaplasia (κ = 0.528 and κ = 0.442), mild agreement was reached for ankylosis and sclerosis, and slight agreement was reached for backfill.

**Figure 3:**
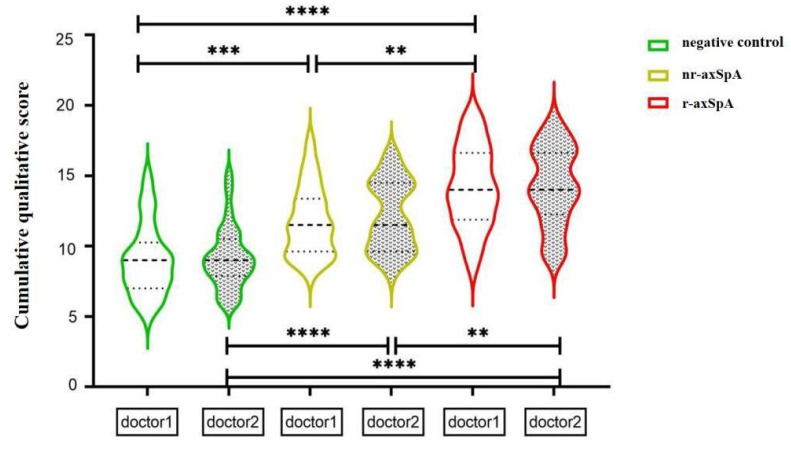
Qualitative and quantitative comparison of the Likert scores of the two readers; comparison of the cumulative scores among the negative control, nr-axSpA, and r-axSpA groups, with significant differences in the average cumulative score among the groups. (****) *p* <0.0001, (***) *p* < 0.001, (**) *p* < 0.01.

The qualitative total score of the two readers could distinguish between the healthy controls and nr-axSpA and r-axSpA groups (Figure 3). The AUC values distinguishing between the healthy control and nr-axSpA groups were 0.7880 and 0.8050 (*p* = 0.838), respectively, with a sensitivity of 0.7500, a specificity of 0.7167, and an accuracy of 0.7339. The AUC values distinguishing between nr-axSpA and r-axSpA were 0.7490 and 0.7110 (*p* = 0.645), respectively, with a sensitivity of 0.6167, a specificity of 0.7344, and an accuracy of 0.6774 (Table 2).

### 3.3. Quantitative TA

**Figure 4:**
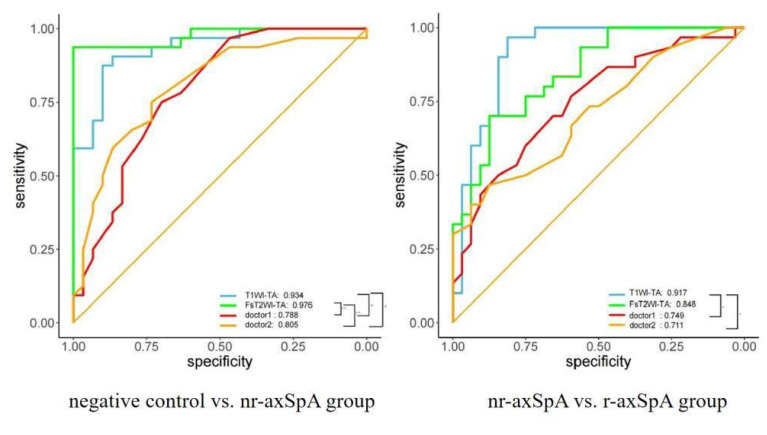
ROC curves of reader 1, reader 2, the T1WI model, and the T2WI model.

The top ten types of features were obtained based on the TA of the T1WI and T2WI sequences. Table 3 shows the odds ratio (OR), the 95% OR, and the *P* value of the characteristic parameters of the four models. Table 4 shows the performance indices of the four models, including *Acc*, *Sen*, *Spe*, *PPV*, *NPV*, and the *AUC*. In distinguishing between the negative control group and the nr-axSpA group, the AUC values of the T1WI and T2WI models were 0.934 and 0.976, respectively (*p* = 0.1838). In distinguishing between the nr-axSpA group and the r-axSpA group, the AUC values of the T1WI and T2WI models were 0.917 and 0.848, respectively (*p* = 0.2592). The comparison of the four TA models showed no significant difference (Figure 4). The qualitative scores of the two readers were compared using the diagnosis performance indices of the TA models. In distinguishing between the negative control group and the nr-axSpA group, the T1WI and T2WI models were both superior to the diagnoses of the two readers (*p* < 0.05) (Figure 4). In distinguishing between the nr-axSpA group and the r-axSpA group, the T1WI model was superior to the diagnoses of the two readers (*p* = 0.023 and *p* = 0.007), and there was no significant difference between the T2WI model and the diagnoses of the readers (*p* = 0.134 and *p* = 0.065) (Figure 4). The relationship between the sensitivity, specificity, and accuracy of the diagnoses made by the two readers and the texture analysis models is visually compared using forest plots, as shown in Supplementary Figure S5: Forest plots of diagnostic performance.

## 4. Discussion

### 4.1. MRI-Based Early Diagnosis

Because MRI has become the most sensitive tool for the detection of early changes in SpA [2], the EULAR guidelines [8] have focused on the identification of BME in the diagnosis of acute sacroiliitis. According to ASAS, to establish the presence of meaningful BME for the diagnosis of sacroiliac arthritis, an oblique coronal image of the sacroiliac joint must show two BME lesions under one articular surface or one obvious BME lesion in two consecutive slices [9]. A qualitative diagnosis depends not only on the clinical background but also on the experience of the reader. Structural lesions of the sacroiliac joint, such as bone erosion, new bone formation, sclerosis and/or fat metaplasia, and ankylosis, are chronic changes after inflammation and only provide supplementary information [10]. BME can also present in normal populations or patients with osteitis condensans or degeneration. Thus, the specificity of a diagnosis based on BME alone is low [11]. Many studies have tried to improve the accuracy of early diagnoses of axSpA by reassessing the clinical history of patients [12] and using contrast-enhanced MRI, yet the outcomes have not been satisfactory [13]. In 2009, the ASAS classification system [1] expanded the criteria, and the sensitivity and specificity of the system are 82.9% and 84.4%, respectively. Because clinicians provide the gold standard of diagnosis, the specificity of the ASAS classification standard is only 84.4%, which is not satisfactory. Therefore, ASAS recognized the importance of the structural changes in axSpA in the diagnosis of sacroiliitis in 2019 [2]. Based on the ASAS recommendations for the diagnosis of SpA, BME is a prerequisite for acute sacroiliitis, and structural lesions, such as bone erosion, new bone formation, sclerosis, and fat metaplasia, should be considered [14]. This study first discussed a qualitative analysis of MR scans for early sacroiliitis diagnosis by two readers, and the diagnostic accuracy was only 70.16%. The agreement between the readers varied greatly for the various signs of sacroiliitis. Considerable agreement was reached for BME (κ = 0.652), moderate agreement was reached for subchondral bone erosion (κ = 0.528) and subarticular fat metaplasia (κ = 0.442), mild agreement was reached for bone buds/ankylosis (κ = 0.339) and subarticular sclerosis (κ = 0.283), and slight agreement was reached for fat metaplasia in an erosion cavity (also known as “backfill”) (κ = 0.115). These results are in line with previous studies [15]. Because the hyperintensity of BME in the acute phase of inflammation on Fs-T2WI is easy to identify, the readers overdiagnosed BME in patients with nonspecific lower back pain. Therefore, there was no significant difference in BME between the control group and the nr-axSpA group for reader 1 (*p* = 1.000). The agreement on subchondral bone erosion between the two readers was moderate. For reader 1, there was a significant difference in the subchondral bone erosion between the nr-axSpA group and the r-axSpA group, yet there was no significant difference between the negative group and the nr-axSpA group. The subchondral bone erosion in the nr-axSpA group was subtle and hypointense and not easily identified. However, the erosion area in the r-axSpA group was large, making it easy to identify.

### 4.2. The Pathology of Sacroiliitis and Routine MR Sequences

There is a layer of articular cartilage attached to the surfaces of the sacroiliac joint, which is hyaline cartilage on the sacrum side and fibrous cartilage on the ilium side, with synovium around the joint. The sacroiliac joint is composed of two portions, the synovial portion and the ligament portion. The synovial portion is anteroinferior to the ilium, and the ligament portion is posterosuperior to the ilium. The synovial portion (i.e., the middle and lower portion) is generally the first place to be subjected to sacroiliitis, and the ligament portion is rarely affected by inflammation. As the inflammatory bony invasion gradually progresses, the joint space becomes blurred, and the articular surface becomes rough, accompanied by BME of the subchondral bone in the early stage. Bone destruction, sclerosis, and osteoporosis may occur in the later stage. Ultimately, the joint space disappears, and the bones fuse.

Non-enhanced MR scans are recommended for the examination of early sacroiliitis, which does not require contrast-enhanced MRI. The conventional scanning position is an oblique coronal scan parallel to the sacroiliac joint’s surface, and the long axis is determined by the center of the S-1 to S-3 vertebral bodies. The conventional sequences include an oblique coronal T1-weighted sequence, an oblique coronal fat-suppressed T2-weighted sequence, an axial T1 sequence, and an axial fat-suppressed T2 sequence. The oblique coronal scan is consistent with the physiological curvature of the sacrum and can reveal the anatomical structures of the sacrum, the ilium, and the sacroiliac joint to the greatest extent. In this study, the coronal T1-weighted sequence and the oblique coronal fat-suppressed T2 sequence, which are the most common and popular sequences in clinical examinations and can cover early inflammatory lesions, were used. According to the principles of fat suppression, Fs-T2WI, SPectral Attenuated Inversion Recovery (SPAIR), short-TI inversion recovery (STIR), Dixon, and ideal techniques can all be used in MR scans of sacroiliac joints. STIR is the most well-known sequence for sacroiliitis and uses inverted radiofrequency pulses to achieve fat signal suppression. Fs-T2WI sequences use spectral fat suppression to eliminate signals from the bone marrow fat and are more sensitive to inflammatory changes in the sacroiliac joint than STIR sequences. In this study, MR scans were obtained using GE 3.0T, using the fs-T2 method. Contrast-enhanced MR does not contribute significantly to the accuracy of early axSpA diagnosis [16]; therefore, EULAR does not recommend the use of intravenous contrast agents to diagnose early sacroiliitis, especially for patients undergoing their first MR examination. In this study, only oblique coronal T1WI and oblique coronal Fs-T2WI sequences were used, and the TA was used to determine the accuracy in early axSpA diagnosis.

### 4.3. TA

TA is a component of radiomics. In 2012, the concept of “radiomics” was first proposed by Dutch scholars and defined as the extraction of many image features from the ROI of an image using a high-throughput method [17]. This method usually consists of image acquisition, image segmentation, feature extraction, and data analysis [18]. TA processes the basic features in the image and achieves non-invasive and effective image processing, thereby providing important information for the diagnosis and treatment of diseases.

Texture is very important visual information. TA is an important component of computer vision. Quantitative TA is an emerging technology that can extract many quantitative features from medical images with a high throughput and convert image data into high-dimensional and mineable data that are invisible to the human eye [19]. In addition, TA can quantify the parameters with high reproducibility. Texture describes the physical properties of the target surfaces, such as the surface roughness. However, the definition of texture is currently still unclear. It is generally believed that texture features refer to grayscale changes in the pixels (or subregions) in an image and are used to describe grayscale or color changes on the target surface. Different physical surfaces have different textures, which are related to the properties of the target itself. TA is widely used in computer vision and image processing. Because some images show regularity at the macro scale but irregularity in the local area, some scholars define texture as an image feature that is irregular locally and regular macroscopically [20]. A substantial amount of visual information can be obtained from textures, and TA can be used to solve many problems in image processing and scientific research.

There are four common TA methods: the statistical method, the structure method, the model method, and the frequency spectrum method [21]. Specifically, the statistical method analyzes the texture information (thickness, uniformity, etc.) based on the grayscale spatial distribution of an image. Because medical images usually have a high grayscale resolution and spatial resolution, statistical methods are often used for the TA of medical images [22]. There are four current research directions for TA: Texture Classification (TC), Texture Retrieval (TR), Texture Segmentation (TS), and Shape from Texture. TC is a method for identifying specific texture regions or patterns in certain texture categories. The TA algorithm extracts certain texture features from each ROI and classifies these features. The statistical methods can be divided into first-order (a single voxel), second-order (two voxels), and high-order (three or more voxels) texture feature analyses. First-order statistics describe the grayscale distribution of the individual pixels in the ROI, which is known as a histogram analysis, and the indices include the average, minimum and maximum, variability, SD, skewness, kurtosis and entropy, heterogeneity, and voxel value percentiles. One limitation of the first-order method is the lack of spatial information. The second-order method is based on the distribution probability of a specified pixel pair, which is often calculated using the GLCM. The GLCM describes the relationship between the intensities of two pixels, and the parameters, such as energy, contrast, correlation, and entropy, are often of statistical significance in medical applications. Thus, the second-order method can be used to supplement first-order statistics. High-order statistics describe the change in intensity or the distribution of homogeneous areas through the difference in the gray levels of adjacent pixels [23].

### 4.4. The Application of TA to Musculoskeletal Imaging Diagnosis

In TAs of medical images, the distributions of the pixels or the voxel gray levels in medical images are obtained using certain image processing technologies, and then many quantitative or qualitative texture features that are invisible to the human eye are extracted. TA is beneficial for the early non-invasive identification of lesions, outcome evaluations, and prognostic predictions. In recent years, TA has been extensively applied in the field of medical imaging, yet the application of TA to the musculoskeletal system is rare. Previous studies have mainly focused on the identification and histological analysis of bone metastases, cartilage tumors, and soft tissue tumors, including the differential diagnosis of benign and malignant cartilage tumors [24] and follow-up treatment of bone metastases [25]. Texture parameters are used to evaluate the degeneration of bones and skeletal muscles [26,27], for example, in quantitative assessments of osteoporosis [28,29]. TA in osteoarthritis (OA) has become a research focus in recent years, with a focus on the articular cartilage and subchondral bone. Studies have shown that TA can indirectly reflect the progress of OA in the articular cartilage and subchondral bone [30]. As a quantitative method, TA not only improves the agreement between readers but can also identify subtle feature differences and therefore improve the specificity of diagnoses. TA can distinguish between and describe different tissues well and improve the differential diagnosis and follow-up treatment of multiple types of bone diseases [31,32]. According to the results of this study, the Fs-T2WI and T1WI models showed similar performances in the differentiation of early sacroiliitis, without significant differences. Compared to the qualitative assessment by the readers, the T1WI model was more stable in the diagnosis of early sacroiliitis (the nr-axSpA stage). Because the difference between nr-axSpA and r-axSpA is mainly due to structural destruction, T1WI sequences can reflect the significant changes between the two better. In addition, because the bone signal after fat suppression is hypointense, these changes are not easily identified in Fs-T2WI sequences. BME in the acute phase shows hyperintensity in Fs-T2WI sequences, and T1WI sequences can highlight structural changes. The structural changes observed on T1WI are more closely associated with disease progression, making these superior sequences for distinguishing nr-axSpA from r-axSpA. Furthermore, the ability of T1WI-TA to capture subtle structural differences results in a higher classification accuracy in this specific context. Therefore, both sequences are basic MR images for sacroiliac joint examinations and have their own features. In clinical practice, in addition to BME on Fs-T2WI, it is necessary to pay attention to structural lesions in T1WI sequences, which are of great significance for the diagnosis of early sacroiliitis.

Quantitative TAs have significant advantages in MR image evaluations over reader evaluations and are more reliable and stable. Integrating texture analysis (TA) into existing diagnostic workflows holds great potential for streamlining radiologists’ assessments and improving diagnostic accuracy. By providing objective and reproducible quantitative data, TA could help reduce interobserver variability and support more consistent evaluations. To date, few studies have used deep-learning-based TAs in the diagnosis of musculoskeletal inflammation. This study, to our knowledge, was the first attempt to apply a TA to studying early changes in the sacroiliac joints.

This study has some limitations. First, this was a retrospective, single-center study. For the purpose of standardization, all patients were scanned using the same scanner using the same coil and scanning protocol. Different scanners, field strengths, and protocols may affect the results of qualitative analyses and TAs. Second, the best scans with no technical flaws, such as motion artifacts, were included because artifacts may have affected the large-scale applicability of the results of this study. Lastly, the results were based on qualitative and quantitative readings by two readers with around 6–7 years of experience because the goal of this study was to represent the majority of readers. Experienced readers may perform better, but the accuracy in sacroiliitis diagnoses is often worse than expected.

## 5. Conclusions

This study demonstrated that a texture analysis (TA) of T1WI and Fs-T2WI sequences of the sacroiliac joints has the potential to assist in the diagnosis of early sacroiliitis by providing quantitative insights that complement qualitative assessments by radiologists. Specifically, the T1WI-TA model exhibited a superior diagnostic performance in distinguishing nr-axSpA from r-axSpA, highlighting its ability to capture subtle structural changes such as fat infiltration and bone erosion. In contrast, the Fs-T2WI-TA model showed a comparable performance to that of the radiologists, primarily reflecting inflammatory activity. These findings suggest that the T1WI-TA model could enhance the early diagnostic efficacy for sacroiliitis, particularly in the nr-axSpA stage, while Fs-T2WI-TA provides a complementary perspective focused on inflammation. Future studies should aim to validate these findings in larger, multicenter cohorts and explore the integration of TA into routine clinical workflows.

## Figures and Tables

**Table 1:** Likert scores of the senior reader (#1).

	Negative Control Group		nr-axSpA Group		r-axSpA Group		Control vs. nr-axSpA	nr-axSpA vs. r-axSpA	Inter-Reader Agreement
	**Left**	**Right**	**Left**	**Right**	**Left**	**Right**	**(Both)**	**(Both)**	**(Both)**
BME	17(0)	17(0)	10(0)	10(2)	13(1)	12(2)	0.009	1.000	0.652
Backfill	16(1)	15(0)	16(1)	19(1)	15(0)	14(0)	0.837	0.153	0.115
Fat Metaplasia	15(1)	16(1)	15(1)	15(1)	13(1)	15(1)	0.580	0.001	0.442
Erosion	17(1)	18(1)	16(1)	21(1)	19(1)	13(1)	0.054	0.001	0.528
Sclerosis	18(2)	18(2)	24(1)	24(1)	15(1)	15(1)	0.009	1.000	0.283
Bone Bud/Ankylosis	30(0)	30(0)	32(0)	32(0)	19(0)	17(0)	1.000	<0.0001	0.339
Total Score	18 ± 5.051		23.47 ± 4.964		28.37 ± 5.864		<0.0001	<0.001	—

Distribution of the qualitative assessment of reader #1 for each parameter, separate per body side and per patient population.

**Table 2:** Diagnostic performance indicators of the two readers.

Group Comparison	Reader	Sensitivity (Sen)	Specificity (Spe)	Accuracy (Acc)	AUC	*P*
	Reader 1	0.7500	0.7000	0.7258	0.7880	
Control vs. nr-axSpA group	Reader 2	0.7500	0.7333	0.7419	0.8050	0.838
	Both Readers	0.7500	0.7167	0.7339	0.7970	
	Reader 1	0.7667	0.5938	0.6774	0.7490	
nr-axSpA vs. r-axSpA group	Reader 2	0.4667	0.8750	0.6774	0.7110	0.645
	Both Readers	0.6167	0.7344	0.6774	0.7300	

**Table 3:** Texture feature parameters.

Model	TA Features	OR (95% CI)	*P*
**Model 1: T1WI-TA**	Original_shape_MajorAxisLength	0.2725 (0.004, 1.2322)	0.126761
	Original_gldm_SmallDependenceEmphasis	0.1608 (0.0326, 0.5100)	0.008062
	Original_glszm_ZoneEntropy	10.1651 (3.4211, 50.5007)	0.0005
**Model 2: T1WI-TA**	Original_glcm_JointEnergy	0.4123 (0.1509, 0.9180)	0.051643
	Original_Shape_SurfaceVolumeRatio	0.1204 (0.0310, 0.3250)	0.000306
	Original_glcm_Imc2	26.1764 (2.0257, 1536.9718)	0.04128
**Model 3: T2WI-TA**	Original_firstorder_Maximum	0.0763 (0.0069, 0.3490)	0.00646
	Original_gldm_SmallDependenceLowGrayLevelEmphasis	0.0625 (0.0021, 0.4248)	0.03019
**Model 4: T2WI-TA**	Original_gldm_LargeDependenceLowGrayLevelEmphasis	2.8098 (1.4046, 6.8188)	0.009720
	Original_shape_VoxelVolume	3.8980 (1.920, 9.5423)	0.000713

**Table 4:** Diagnostic performance indices of the TA model.

Models	Acc	Sen	Spe	PPV	NPV	AUC	95% CI
**Model 1: T1WI-TA**	0.8871	0.8750	0.9000	0.9032	0.8710	0.934	0.869–0.999
(negative control vs. nr-axSpA)							
**Model 2: T1WI-TA**	0.8871	0.9667	0.8125	0.8286	0.9630	0.917	0.843–0.991
(nr-axSpA vs. r-axSpA)							
**Model 3: T2WI-TA**	0.9677	0.9375	1.0000	1.0000	0.9375	0.976	0.937–1.014
(negative control vs. nr-axSpA)							
**Model 4: T2WI-TA**	0.7903	0.7000	0.8750	0.8400	0.7568	0.848	0.749–0.947
(nr-axSpA vs. r-axSpA)							

## Data Availability

The dataset can be made available by the corresponding author upon reasonable request.

## Supplementary Materials

The following supporting information can be downloaded at https://www.mdpi.com/article/10.3390/diagnostics15020209/s1. Supplementary Data S1: Dataset Description; Table S1: Detailed Sample-Wise Distribution of the Dataset; Figure S1: Dataset Sample Images; Data S2: The radiological classification standards are as follows; Data S3: Consistency Evaluation of Feature Extraction; Table S2: MRI Scanning Parameters; Table S3: Likert Score by the Junior Reader (#2); Figure S2: Lesion Segmentation; Figure S3: Oblique Coronal Image; Figure S4: Lesions of Sacroiliitis; Figure S5: Forest Plots of Diagnostic Performance.
