# Supplementary material for: Differentiation of Early Sacroiliitis Using Machine-Learning- Supported Texture Analysis
Source: Diagnostics (Basel). 2025 Jan 17;15(2):209. doi: 10.3390/diagnostics15020209 (PMC11763746; doi:10.3390/diagnostics15020209)
Supplement: Supplementary file 1 [file diagnostics-15-00209-s001.zip › diagnostics-3312752-supplementary.pdf]

Supplementary Materials

Supplementary Materials

Supplementary Data S1: Dataset Description

Total Cases: 92 cases, including:

- **62 cases of axial spondyloarthritis (axSpA):**
  - **32 cases of non-radiographic axial spondyloarthritis (nr-axSpA):** Bilateral sacroiliac joints DR or CT grade < II, or unilateral sacroiliac joint DR or CT grade < III.
  - **30 cases of radiographic axial spondyloarthritis (r-axSpA):** Bilateral sacroiliac joints DR or CT grade II, or unilateral sacroiliac joint DR or CT grade III.
- **30 cases in the control group:** Diagnosed with normal sacroiliac joints during clinical follow-up.

Demographics:

- AxSpA group: 39 males and 23 females, aged 18–44 years (mean 31.18±8.80 years).
- Control group: 17 males and 13 females, aged 18–44 years (mean 31.00±8.63 years).

Inclusion Criteria: The axSpA group was diagnosed based on the 2009 ASAS classification criteria, and all patients underwent 3T MRI, X-ray, and/or CT within one week of MRI.

Exclusion Criteria: Cases with unilateral or bilateral grade IV sacroiliitis, trauma, infection, prior sacroiliac joint surgery, and other confounding conditions were excluded.

Supplementary table S1:Detailed Sample-Wise Distribution of the Dataset

Dataset	Normal (Control Group)	nr-axSpA (Patients)	r-axSpA (Patients)	Total
Cases	30	32	30	92
Sacroiliac Joint Images	1180	1280	1168	3628

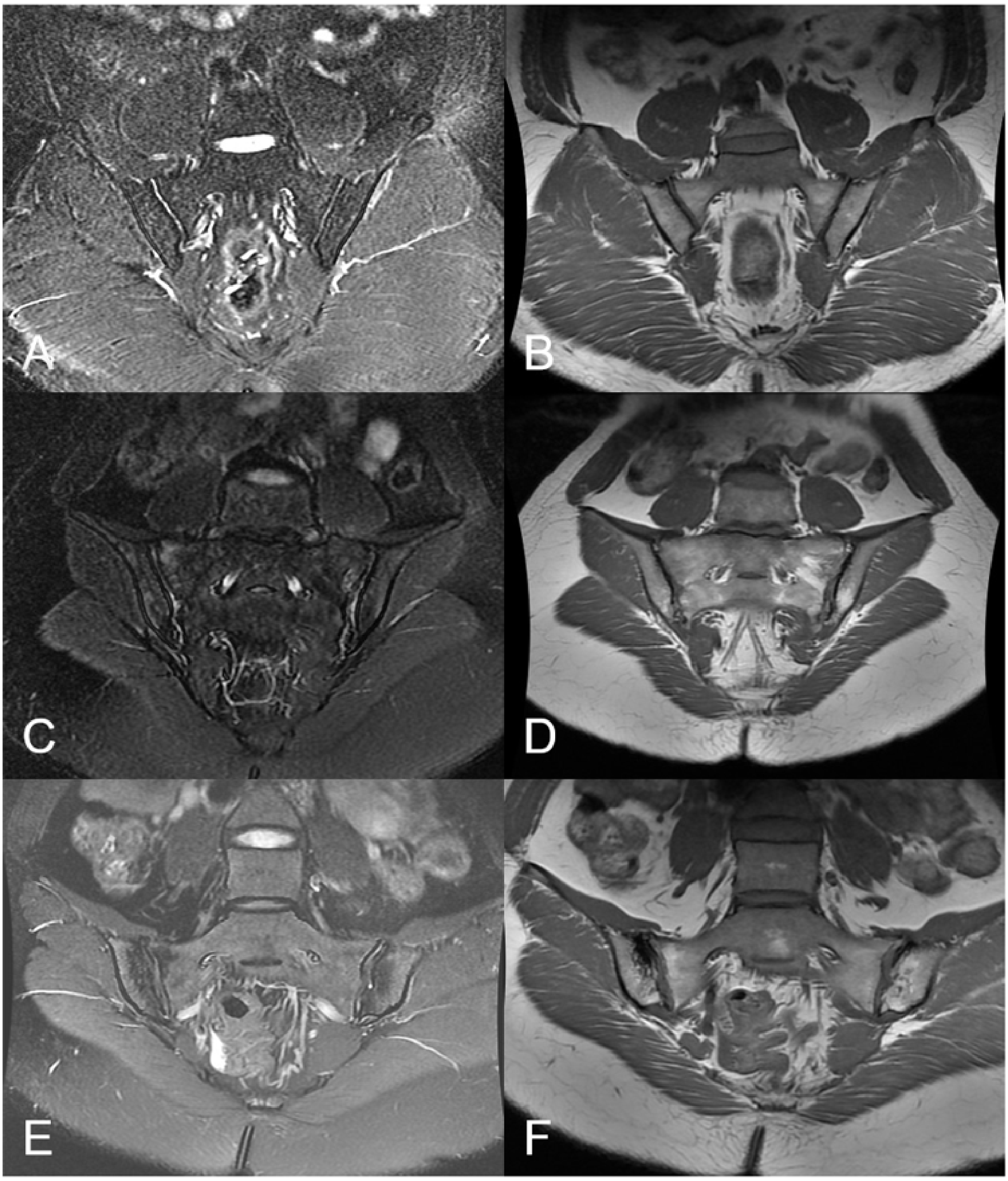

Supplementary Figure S1:Dataset Sample Images
(A, B) ob-FsT2WI Coronal and ob-T1WI Coronal images of the control group; (C, D) ob-FsT2WI Coronal and ob-T1WI Coronal images of nr-axSpA; (E, F) ob-FsT2WI Coronal and ob-T1WI Coronal images of r-axSpA.

Supplementary Data S2: The radiological classification standards are as follows:

X-ray classification: Grade 0: normal; Grade I: suspicious; Grade II: mild, localized joint erosion and hardening, no change in joint space; Grade III: moderate, bone erosion and hardening of the articular surface, and widening/narrowing or partial ankylosis of the joint space; Grade IV: severe, complete bony ankylosis of the joint.

CT classification: Grade 0: normal joints or slightly blurred articular surface contours; Grade I: osteoporotic joint axis, mild subchondral erosion, blurred articular surface contours, and normal joint space; Grade II: blurred articular surface contours, subchondral bone destruction, osteoporosis and joint hardening, and normal joint space; Grade III: moderate destruction of subchondral bone, diffuse sclerosis, brush-like articular surface, uneven joint space, and partial ankylosis; Grade IV: whole joint destruction, sclerosis and osteoporosis, and complete joint ankylosis.

Supplementary Data S3: Consistency Evaluation of Feature Extraction

The Dice Similarity Coefficient value for the segmentations performed by the two radiologists was 0.81. Two radiologists extracted ROIs with an inter-observer ICC range of 0.73-0.92 and an intra-observer ICC range of 0.91-0.95. To ensure the reproducibility of radiomics features, both inter-observer and intra-observer ICC values were required to exceed 0.75. Features with intra-observer and inter-observer ICC values below 0.8 were excluded, leaving 96 features from T1WI images and 90 features from FsT2WI images.

Supplementary table S2: MRI Scanning Parameters

**Parameter**	**ob-T1WI Axial**	**ob-T1WI Coronal**	**ob-FsT2WI Axial**	**ob-FsT2WI Coronal**
**TR** (ms)	500	720	3920	3675
**TE** (ms)	9.8–29.5	7.3–22.0	85	85
**FOV** (mm)	432 × 432	456 × 456	320 × 224	320 × 224
**Matrix**	352 × 192	320 × 224	320 × 224	320 × 224
**Slices (n)**	20	21	19-20	19-20
**Slice Thickness (mm)**	5.0	4.0	5.0	4.0
**Slice Spacing (mm)**	1.0	1.0	1.0	1.0

Supplementary table S3:Likert Score by the Junior Reader (#2)

**Parameter**	**Control Left (Right)**	**nr-axSpA Left (Right)**	**r-axSpA Left (Right)**	**Control vs nr-axSpA**	**nr-axSpA vs r-axSpA**
**BME**	15(1) 18(0)	16(0) 11(1)	17(1) 22(1)	0.019	0.025
**Backfill**	16(0) 25(0)	25(0) 20(1)	20(1) 15(0)	0.234	0.673
**Fat Metaplasia**	14(1) 14(1)	15(0) 15(0)	11(2) 11(2)	0.003	<0.001
**Erosion**	15(1) 14(0)	17(1) 17(1)	15(1) 12(2)	0.002	0.015
**Sclerosis**	13(1) 12(1)	15(1) 17(2)	12(2) 11(1)	0.002	0.013
**Bone Bud/Ankylosis**	18(0) 29(0)	29(0) 30(0)	17(1) 18(1)	0.75	<0.001
**Total Score**	16 ± 2.613	24.97 ± 5.682	26.05 ± 7.927	<0.0001	<0.01

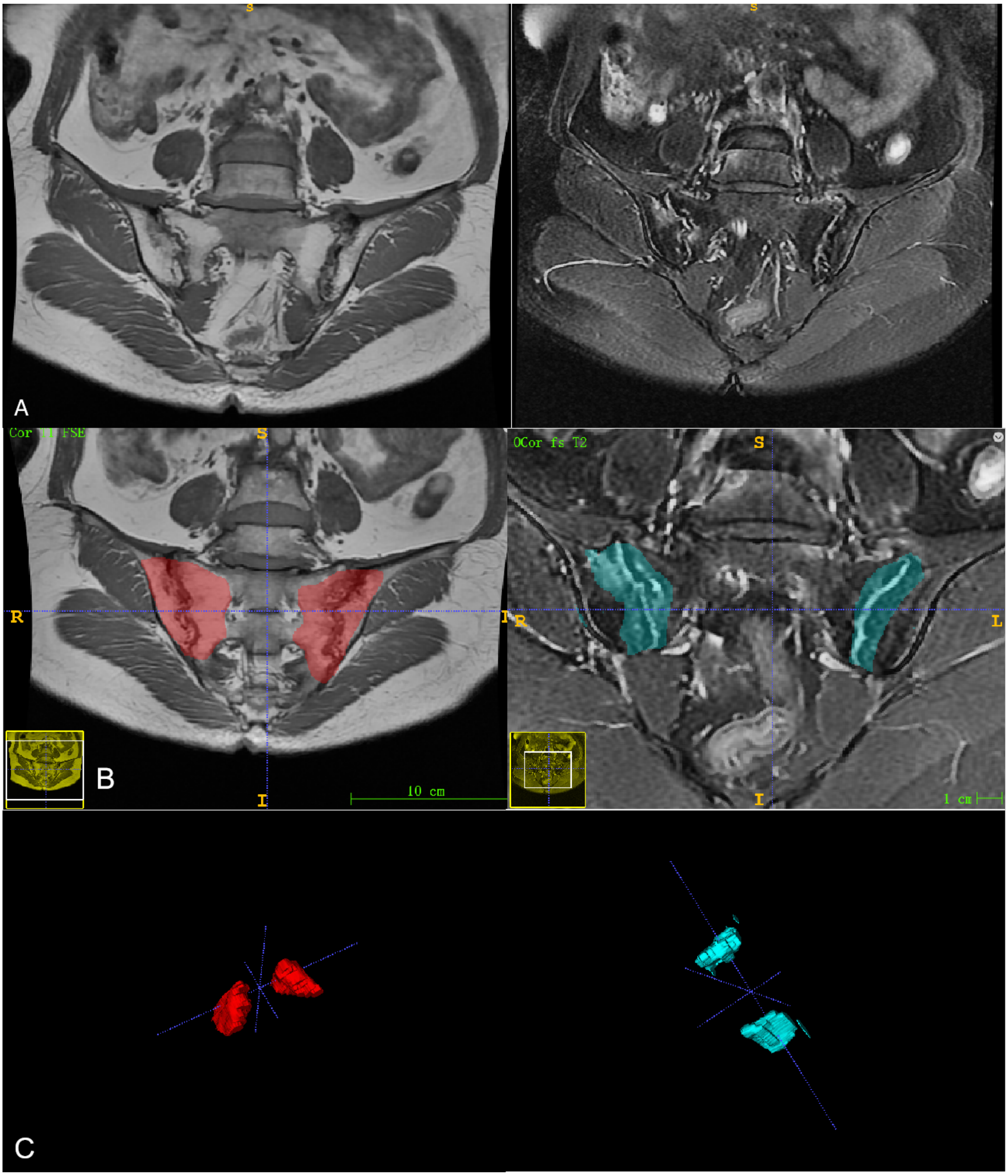

Supplementary Figure S2: Lesion Segmentation
(A) Lesion of SIJ; (B) Segmentation on the oblique coronal slice; (C) Three-dimensional volumetric reconstruction.

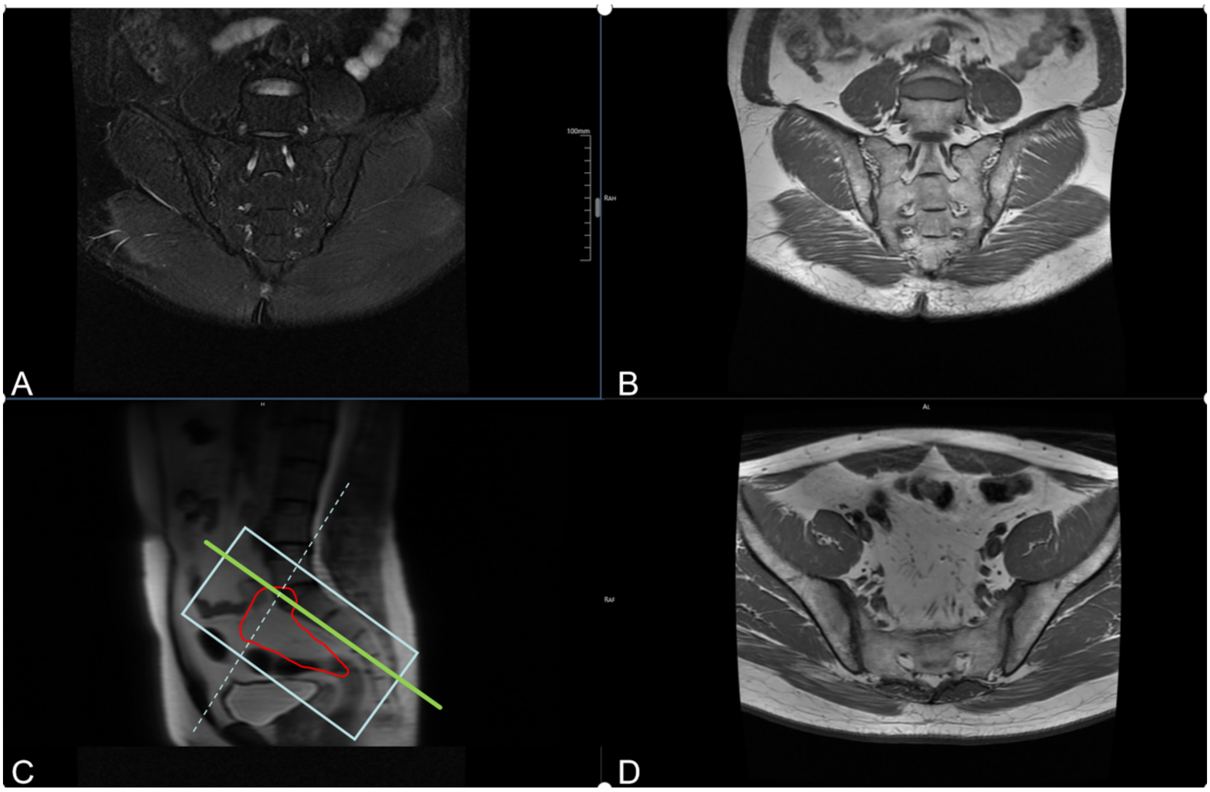

Supplementary Figure S3: Oblique Coronal Image
(A) Oblique coronal Fs-T2WI; (B) Oblique coronal T1WI; (C) Red lines: projection area of the sacroiliac joint; green lines: orientation of the oblique coronal positioning line; blue box: oblique coronal scanning range; blue dashed lines: oblique axial direction; (D) Oblique axial T1WI.

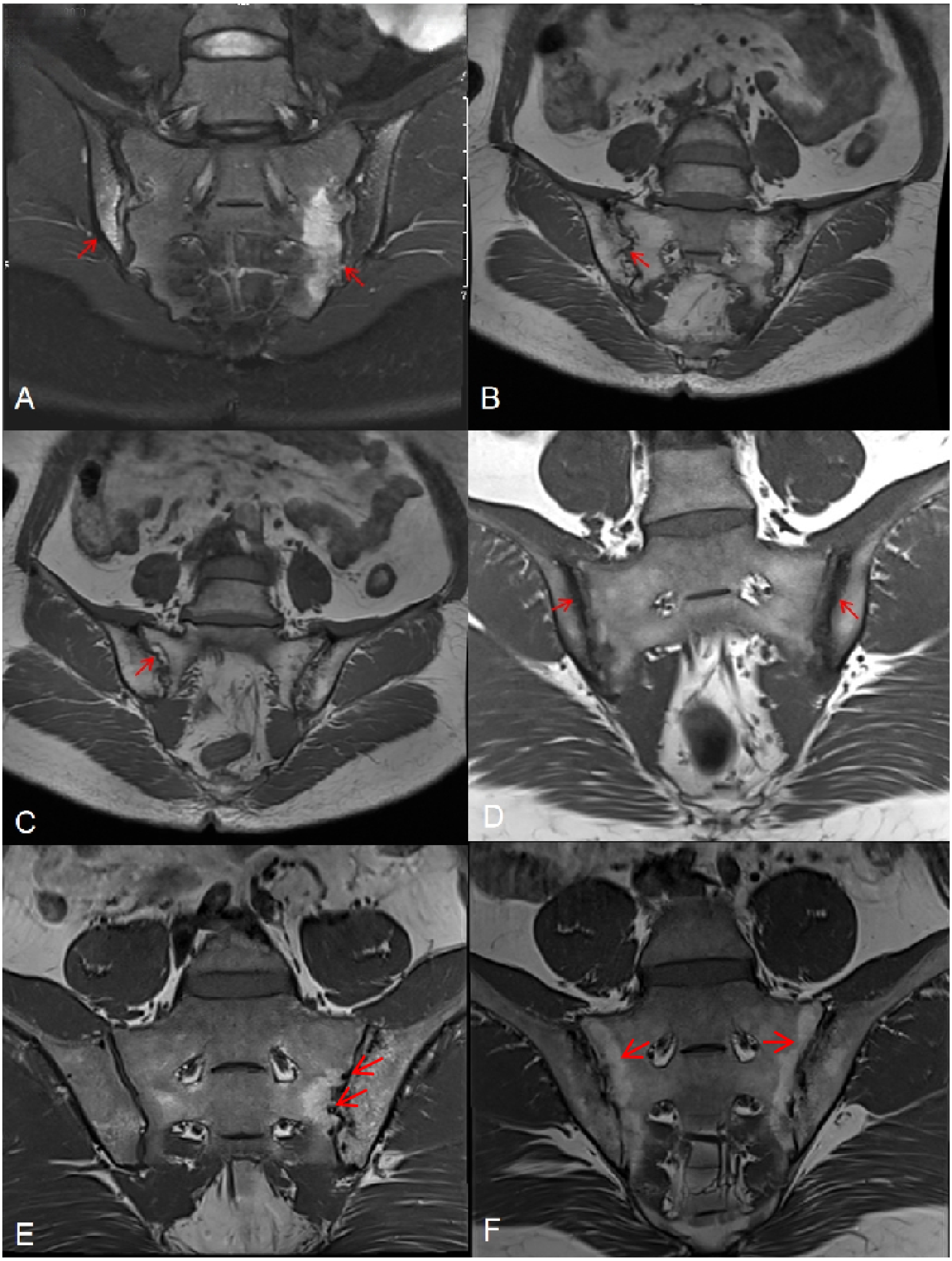

Supplementary Figure S4: Lesions of Sacroiliitis
(A) BME; (B) Bone Bud/Ankylosis; (C) Backfill; (D) Sclerosis; **(E) Erosion; (F) fat metaplasia.**

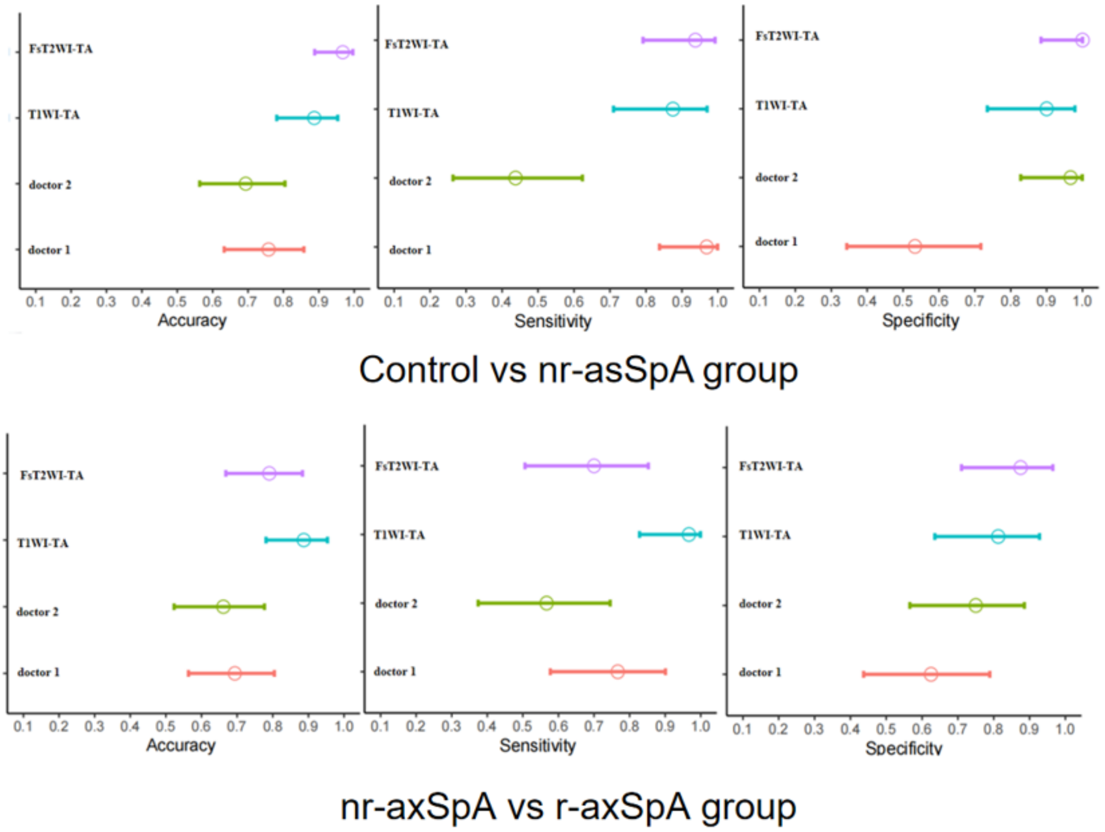

Supplementary Figure S5: Forest Plots of Diagnostic Performance
Forest plots illustrating the diagnostic performance (accuracy, sensitivity, and specificity) of the two readers (doctor 1 and doctor 2) and the texture analysis models (T1WI-TA and FsT2WI-TA).
